# DNA Methylation and Normal Chromosome Behavior in Neurospora Depend on Five Components of a Histone Methyltransferase Complex, DCDC

**DOI:** 10.1371/journal.pgen.1001196

**Published:** 2010-11-04

**Authors:** Zachary A. Lewis, Keyur K. Adhvaryu, Shinji Honda, Anthony L. Shiver, Marijn Knip, Ragna Sack, Eric U. Selker

**Affiliations:** 1 Institute of Molecular Biology, University of Oregon, Eugene, Oregon, United States of America; 2 Friedrich Miescher Institute for Biomedical Research, Basel, Switzerland; University of Cambridge, United Kingdom

## Abstract

Methylation of DNA and of Lysine 9 on histone H3 (H3K9) is associated with gene silencing in many animals, plants, and fungi. In *Neurospora crassa*, methylation of H3K9 by DIM-5 directs cytosine methylation by recruiting a complex containing Heterochromatin Protein-1 (HP1) and the DIM-2 DNA methyltransferase. We report genetic, proteomic, and biochemical investigations into how DIM-5 is controlled. These studies revealed DCDC, a previously unknown protein complex including DIM-5, DIM-7, DIM-9, CUL4, and DDB1. Components of DCDC are required for H3K9me3, proper chromosome segregation, and DNA methylation. DCDC-defective strains, but not HP1-defective strains, are hypersensitive to MMS, revealing an HP1-independent function of H3K9 methylation. In addition to DDB1, DIM-7, and the WD40 domain protein DIM-9, other presumptive DCAFs (DDB1/CUL4 associated factors) co-purified with CUL4, suggesting that CUL4/DDB1 forms multiple complexes with distinct functions. This conclusion was supported by results of drug sensitivity tests. CUL4, DDB1, and DIM-9 are not required for localization of DIM-5 to incipient heterochromatin domains, indicating that recruitment of DIM-5 to chromatin is not sufficient to direct H3K9me3. DIM-7 is required for DIM-5 localization and mediates interaction of DIM-5 with DDB1/CUL4 through DIM-9. These data support a two-step mechanism for H3K9 methylation in Neurospora.

## Author Summary

Eukaryotic genomes are composed of distinct structural and functional domains marked by various covalent modifications of histone proteins and, in some organisms, by methylation of cytosine bases in DNA. Gene-rich euchromatin exists in a relatively open conformation, facilitating DNA transactions such as transcription, whereas the gene-poor heterochromatin is more condensed and is a poor substrate for DNA–based transactions. Heterochromatin promotes genome stability by silencing transposons and may be essential for proper centromere function. DNA methylation is a common feature of heterochromatin in eukaryotes, including the filamentous fungus *Neurospora crassa*, which has served as a model system to elucidate the control of DNA methylation. All DNA methylation in Neurospora depends on histone H3 lysine-9 (H3K9) methylation, which is recognized by a complex of HP1 (Heterochromatin Protein 1) and the DNA methyltransferase, DIM-2. An important open question is what controls the H3K9 methyltransferase, DIM-5. We report the genetic and proteomic identification of a DIM-5 protein complex, DCDC, and demonstrate that it includes five proteins essential for H3K9 methylation, DNA methylation, proper chromosome segregation, and resistance to DNA damaging agents. In addition, we report molecular and genetic analyses revealing a hierarchy of protein interactions within DCDC.

## Introduction

Methylation of selected cytosines in DNA is a prototypical epigenetic process found in many eukaryotes. DNA methylation has been implicated in embryonic development, genome imprinting, X chromosome inactivation, transposon silencing and gene regulation [1]–[5]. Conversely, abnormal DNA methylation has been associated with disease in humans, developmental defects in plants and growth defects in Neurospora [6]–[8]. Although some functions of DNA methylation have been identified, its regulation is not completely understood. The filamentous fungus *Neurospora crassa* has emerged as an excellent model system to elucidate the control of DNA methylation. In this organism, DNA methylation is found almost exclusively associated with relics of a genome defense system, RIP (repeat-induced point mutation) [9], [10]. The RIP machinery detects and mutates duplicate sequences during the sexual cycle, littering each copy with C to T transition mutations [11], [12]. Notably, the resulting A:T-rich sequences tend to be potent signals for *de novo* DNA methylation [9], [13], [14]. Our previous genetic studies revealed that all DNA methylation in Neurospora is dependent on a single DNA methyltransferase, DIM-2, (named for defective in DNA methylation) [15], an H3K9 methyltransferase (KMT), DIM-5 [16], Heterochromatin Protein-1 (HP1) [17] and DIM-7, a protein that interacts with DIM-5 [18]. The demonstration that DNA methylation depends on H3K9 methylation in Neurospora was followed quickly by findings that histone methylation is also critical for some DNA methylation in both plants and animals [19]–[21], suggesting that components of the DNA methylation pathway of Neurospora may be conserved in higher eukaryotes.

DIM-5 catalyzes *tri-*methylation of H3K9 (H3K9me3), which is recognized and bound by a complex of HP1 and DIM-2 [17], [22], [23]. Direct interaction of the chromo shadow domain of HP1 with a pair of PXVXL-like motifs in DIM-2 is essential for DNA methylation and does not depend on H3K9me3 [23]. In Neurospora, H3K9me3, HP1 and DNA methylation co-localize at RIP'd sequences and together define domains of heterochromatin at centromeres, telomeres and dispersed RIP'd regions throughout the genome [9]. Notably, the distribution of H3K9me3 is unaffected in the *dim-2* mutant and is also independent of HP1 at nearly all heterochromatin domains [9], [23]. Efficient *de novo* DNA methylation is observed following depletion and subsequent re-introduction of H3K9 methylation [9]. Thus, RIP'd DNA directs H3K9 methylation and subsequent DNA methylation primarily through a unidirectional pathway.

Here we report that purification of DIM-5-associated proteins, in conjunction with genetic studies based on a powerful new selection for mutants defective in DNA methylation [18], revealed a multi-subunit complex, DCDC, that directs histone methylation in Neurospora. All five core members of the complex, DIM-7, DIM-8 (DDB1), DIM-9 and CUL4, are essential for H3K9 and DNA methylation but DIM-7 is uniquely required to target DIM-5 to heterochromatin domains and is also required to connect DIM-5 to the DCAF (DDB1/CUL4 Associated Factor), DIM-9.

## Results

### Identification of *dim* genes essential for DNA and H3K9 methylation

**Figure 1:**
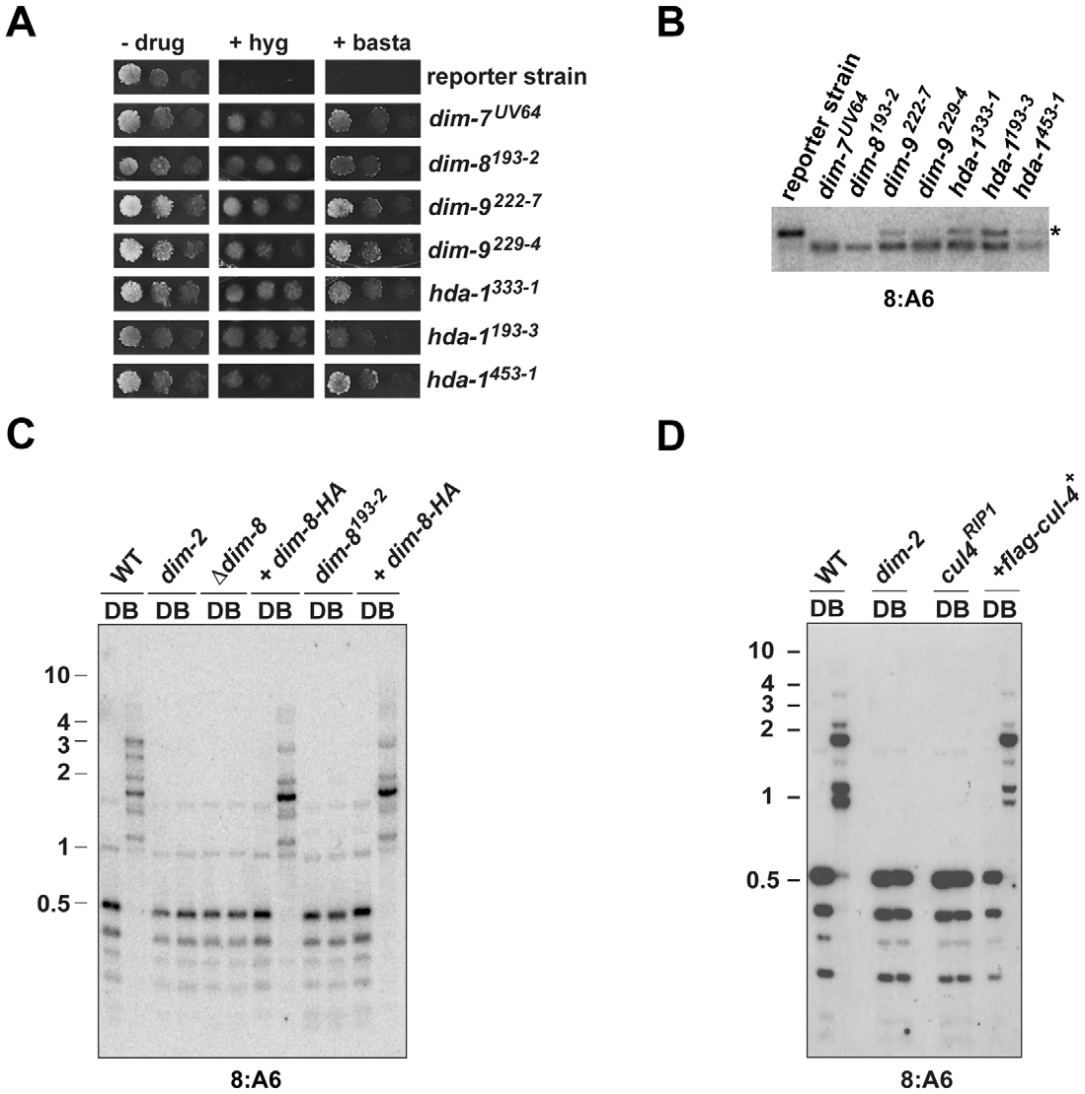
CUL4 and DDB1 are essential for DNA methylation. (A) Suspensions of 10^4^, 10^3^ or 10^2^ conidia of the indicated strains were spot-tested on media with or without basta or hygromycin. (B) Southern hybridization with a probe corresponding to the normally methylated 8:A6 region was performed using genomic DNA digested with *Bam*HI and *Eco*RI from methylation-positive (reporter strain) and -negative (*dim-7^UV64^*) control strains, as well as putative *dim* mutants. The asterisk indicates the expected position of the methylated DNA fragment (C, D) Southern hybridizations with a probe corresponding to the normally methylated 8:A6 region was performed using genomic DNA from the indicated strains digested with the cytosine-methylation-sensitive *Bfu*CI (B) and –insenstive *Dpn*II (D) endonucleases. The numbers at the left of each blot indicate the size, in kilobases, and position of molecular weight markers.

Neurospora mutants defective in DNA methylation, such as *dim-2* [24] and *dim-5* [16], were initially identified by laborious screening, by happenstance or, later, by reverse genetics [17]. Because there was no indication that the genome had been thoroughly searched for non-essential *dim* genes, we recently developed a dual reporter strain harboring methylated copies of drug-resistance genes (*bar* and *hph*, conferring resistance to basta and hygromycin, respectively) that could be used to select for *dim* mutants [18]. We decided to use this strain for an insertional mutagenesis, reasoning that the insertions could be used as tags to quickly identify the *dim* genes (see Methods). We identified eleven candidate insertional mutants, which were basta- and hygromycin-resistant, exhibited reduced or no DNA methylation at the normally methylated 8:A6 region [10] and gave rise to Dim^−^ progeny in sexual crosses (Figure 1A, 1B and data not shown). Curiously, genetic analyses revealed that the insertion cassette was not responsible for the Dim^−^ phenotype of 10 of the 11 mutants (data not shown). The single potential insertional mutant strain, which we named *dim-8*, displayed an apparent complete loss of DNA methylation (Figure 1B, 1C). Using inverse PCR, we found that the insertion cassette had integrated within NCU06605, a gene encoding the Neurospora homolog of DDB1 (Damaged DNA Binding Protein-1; see Text S1). To confirm that the insertion into NCU06605 was indeed responsible for the Dim^−^ phenotype, we tested a NCU06605 knockout strain available from the Neurospora Genome Project [25]. Like our *dim-8* strain, the NCU06605 knockout strain displayed an apparent complete loss of DNA methylation (Figure 1C). We next tested for complementation of the methylation defects of the *dim-8* and NCU06605 knockout strains by introducing a 3XHA-tagged copy of the gene. DNA methylation was successfully restored in both strains (Figure 1C), confirming that disruption of NCU06605 was responsible for the methylation defect of the *dim-8* strain. We therefore refer to NCU06605 as *dim-8* and its protein product as DDB1.

Two additional mutants mapped to LGII and comprised a novel complementation group, which defined the *dim-9* gene. The identity of *dim-*9 was revealed following purification and identification of DIM-5-associated proteins (see below). Complementation analyses also revealed that three additional strains represent new alleles of *histone deacetylase-1*, which we already knew is required for normal levels of DNA methylation [26].

### CUL4 is essential for methylation of DNA and H3K9

DDB1 is known to interact with Cullin4 (CUL4) to form the core of an E3 ubiquitin ligase [27]. We utilized RIP to create a *cul4* mutant strain and found that DNA methylation was abolished in this strain (Figure 1D, Figure S1). To verify that disruption of *cul4* was responsible for the loss of DNA methylation, we introduced a FLAG-tagged copy of CUL4 (FLAG-CUL4; see Text S1). DNA methylation was restored in this strain, demonstrating that like DDB1, CUL4 is essential for DNA methylation (Figure 1D, Figure S1).

**Figure 2:**
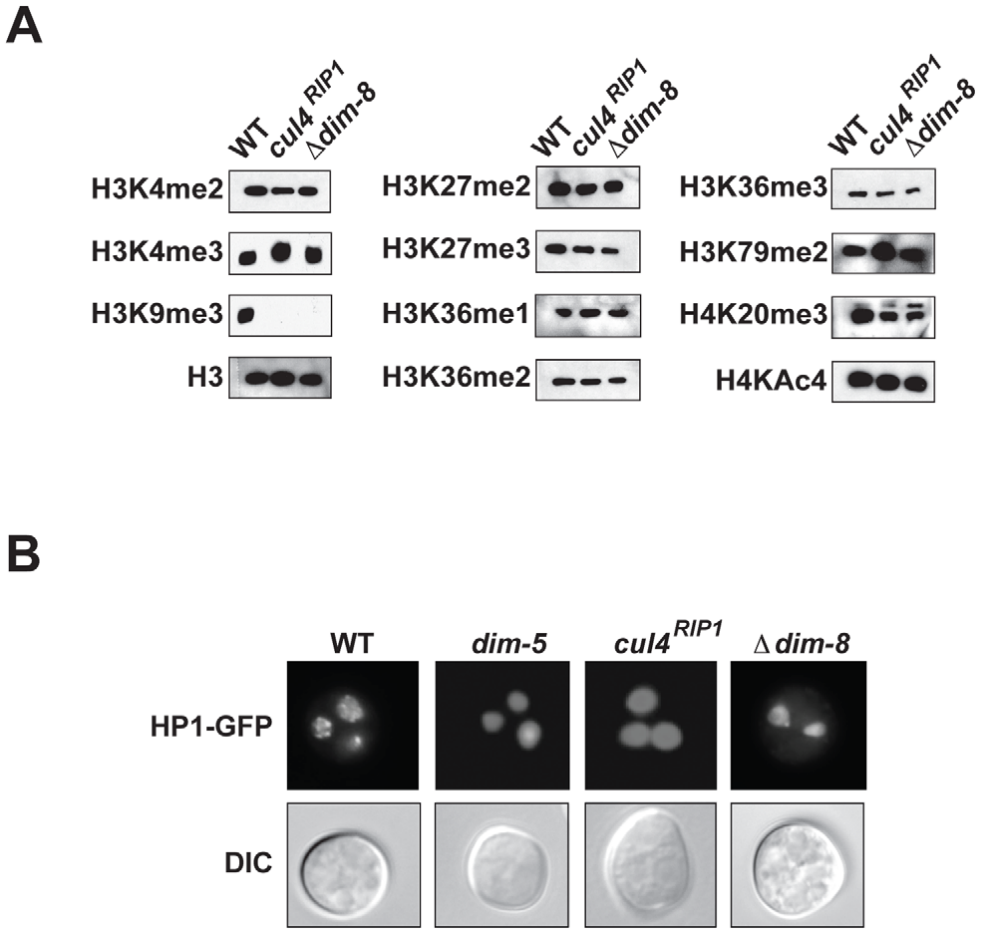
CUL4 and DDB1 are essential for H3K9 methylation. (A) Nuclear extracts from wildtype, *dim-8* and *cul4* were subjected to western blotting using the indicated antibodies specific for various modified histone proteins or for unmodified H3. (B) The distribution of HP1-GFP is shown in multinucleate conidia for wildtype, *dim-5*, *dim-8* and *cul4* strains (DIC; differential interference contrast).

In *Schizosaccharomyces pombe*, CUL4 and the divergent DDB1 homolog Rik1 are essential for H3K9 methylation at heterochromatin domains [28], [29]. Although Neurospora DDB1 is more similar to DDB1 homologues than to Rik1 (49% similar to *Arabidopsis* DDB1A; 46% similar to *S. pombe* Ddb1; 45% similar to human DDB1; 39% similar to *S. pombe* Rik1; determined by BLAST searches queried with Neurospora DDB1), the similarity between these proteins suggested that they could perform similar functions. We therefore tested if CUL4 and DDB1 are required for H3K9 methylation in Neurospora, which we already knew is essential for DNA methylation in this organism [16]. Western blots revealed that H3K9me3 was completely abolished in the *cul4^RIP1^* and Δ*dim-8* mutant strains (Figure 2A). Recent work with mammalian cells revealed that CUL4 and DDB1 are important for methylation of additional residues on H3, including H3K4 and H3K27 [30]. We therefore examined the levels of H3K4me, H3K27me, H3K36me, H3K79me and H4K20me in these mutant strains. Western blots revealed that only H3K9 methylation was affected in the *cul4* and *dim-8* strains (Figure 2A). HP1 localization to heterochromatic foci within the nucleus is dependent on H3K9me3 in Neurospora [17]. As expected, HP1 was mislocalized in the *cul4* and *dim-8* strains, consistent with a complete loss of H3K9 methylation (Figure 2B).

### Identification of DIM-5–associated proteins

**Figure 3:**
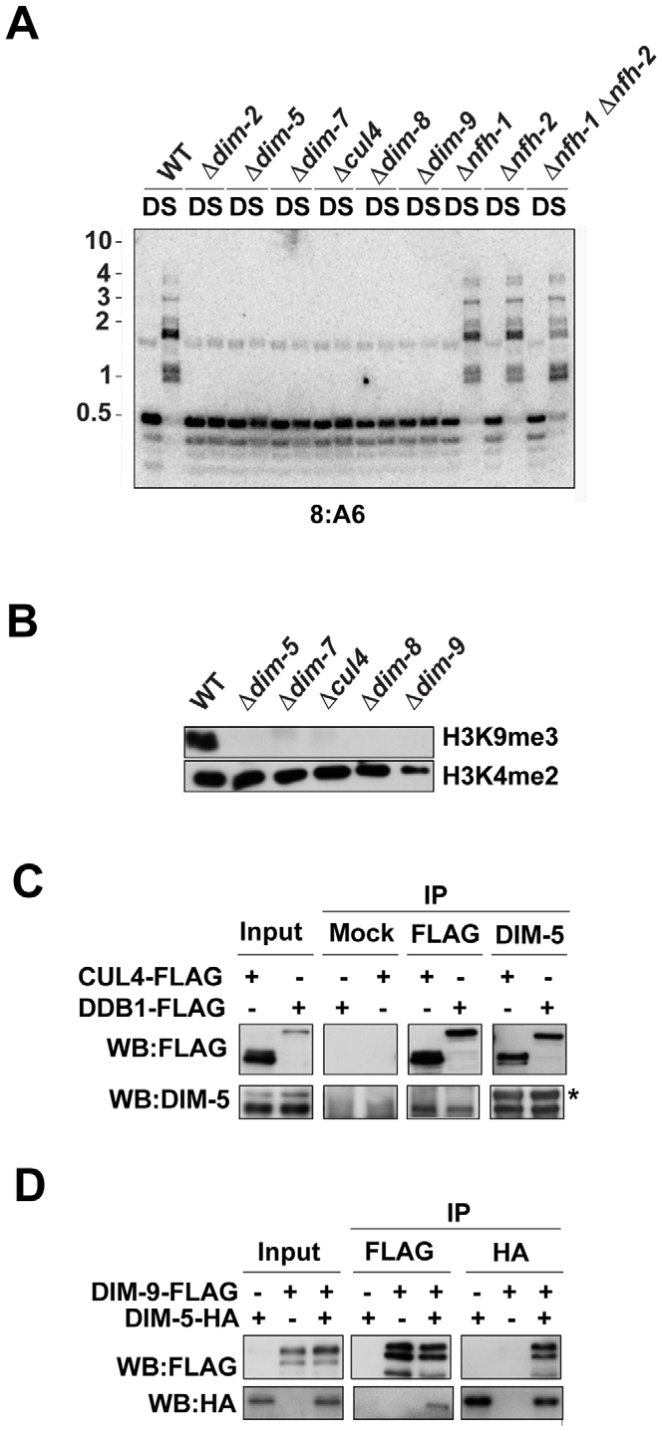
DIM-5 interacts with CUL4, DDB1, and DIM-9. (A) Southern hybridization with a probe corresponding to the normally methylated 8:A6 region was performed using genomic DNA from the indicated strains digested with the cytosine-methylation-sensitive *Bfu*CI (B) and –insensitive *Dpn*II (D) endonucleases. (B) Histones extracted from wildtype, *dim-5*, *dim-7*, *dim-8*, *cul4 and dim-9* were subjected to western blotting using antibodies to H3K9me3 and H3K4me2 as indicated. (C) Immunoprecipitation experiments were performed using extracts from strains expressing FLAG-CUL4 or DDB1-FLAG (+). The input fraction, the α-FLAG immunoprecipitate (IP:αFLAG), the α-DIM-5 immunoprecipitate (IP: α-DIM-5) and the mock immunoprecipitate (IP: mock) were subject to western blotting and probed with the α-FLAG or α-DIM-5 antibodies as indicated (WB). The asterisk indicates a non-specific cross-reacting band. (D) Immunoprecipitation experiments were performed using extracts from strains expressing DIM-9-FLAG, DIM-5-HA, or both (indicated by + or −). The input fraction, the α-HA immunoprecipitate fraction (IP:α-HA) and the α-FLAG immunoprecipitate fraction (IP:α-FLAG) were subjected to western blotting and probed with the α-FLAG or α-HA antibodies as indicated (WB).

In addition to the genetic approach described above, we also employed biochemical approaches to identify DIM-5-associated proteins. We engineered a strain expressing DIM-5 fused to a HAT-FLAG tandem affinity tag [31] and used this in a two-step purification of DIM-5. The purified material was then analyzed by mass spectrometry. We identified peptides covering 25% of DIM-5, 25% of the previously characterized DIM-5-interacting protein DIM-7 [18], 11% of CUL4 and 13% of DDB1 (Table S1). Other potentially relevant proteins were also identified. CUL4/DDB1 complexes are known to interact with DCAFs that have WD40 domains and serve as substrate specificity factors [32], [33]. We identified peptides covering 8% of a WD40 domain-containing protein encoded by NCU01656 (Table S1). This gene resides on LGII, which raised the possibility that it was the unidentified *dim-9* gene revealed in our mutant hunt. To test this possibility, we sequenced NCU01656 from the *dim-9^222-7^* strain. A 120 bp deletion near the C-terminus was found, which would remove amino acids 1178 to 1217 from the predicted protein (XP_956278.2), suggesting that this gene was *dim-9*. We next introduced a wildtype copy of the NCU01656 gene into the *dim-9* strain to test for complementation. DNA methylation was restored (Figure S2), demonstrating that mutations in NCU01656 are indeed responsible for loss of methylation in the *dim-9* strains. We therefore refer to NCU01656 as *dim-9* and the encoded protein as DIM-9. This gene had been replaced with an *hph* cassette as part of the Neurospora genome project [25] but homokaryotic strains had not been successfully isolated, suggesting that DIM-9 might be essential for viability or meiosis. To examine these possibilities, we crossed the heterokaryotic *dim-9* replacement strain to a *Sad-1* strain to prevent meiotic silencing by unpaired DNA [34] and isolated hygromycin-resistant progeny. We were able to obtain homokaryotic *dim-9* knock-out progeny, indicating that the gene is not essential for viability. Southern blot analyses revealed that DIM-9 is essential for DNA methylation, like DIM-5, DIM-7, DDB1 and CUL4 (Figure 3A). Similarly, western blots revealed that the *dim-9* knock-out strain displayed an apparent complete loss of H3K9me3 (Figure 3B).

In addition to DIM-9, we identified peptides covering 19% of one Neurospora 14-3-3 domain-containing protein and 12% of another such protein (Table S1), which together represent the only two genes encoding 14-3-3 domain proteins in the *N. crassa* genome [35]. We refer to these previously uncharacterized genes as *Neurospora fourteen-three-three homolog-1* (*nfh-1*; NCU3300) and *nfh-2* (NCU02806). An *S. pombe* 14-3-3 protein was recently shown to interact with the Clr4 KMT and to function in heterochromatin formation [36]. We were interested to determine if one or both Neurospora 14-3-3 protein(s) is/are required for heterochromatin formation. Because knockout strains lacking *nfh-2* were not available, we replaced the *nfh-2* gene with the selectable *bar* gene [37] by targeted gene replacement [38]. Southern analysis of this and an *nfh-1* knockout strain obtained from the Neurospora genome project revealed normal DNA methylation in both *nfh* mutant strains (Figure 3A). The predicted amino acid sequences of NFH-1 and NFH-2 are similar, suggesting that these proteins may perform redundant functions. To test this, we created an *nfh-1*, *nfh-2* double mutant strain. The double mutant exhibited severe growth defects but we were able to obtain enough tissue to assess DNA methylation. In contrast to the results obtained for *dim-5*, *dim-7*, *dim-8*, *cul4* and *dim-9* strains, Southern blots revealed only a mild loss of DNA methylation in the *nfh-1*, *nfh-2* strain (Figure 3A). Although we were unable to obtain enough tissue from the *nfh* double mutant to isolate histones, the persistence of DNA methylation predicts that H3K9 methylation is present in this strain.

We previously showed that DIM-7 interacts with DIM-5 *in vivo* [18]. To verify that DDB1, CUL4 and DIM-9 also interact with DIM-5, we performed coimmunoprecipitation (CoIP) experiments with strains expressing epitope-tagged proteins. We expressed a C-terminal, 3XFLAG-tagged DDB1 (DDB1-FLAG) from its native locus and similarly used the FLAG-CUL4 strain described above. Following immunoprecipitation with anti-FLAG antibodies or anti-DIM-5 antibodies, western blots revealed both DIM-5 and the expressed DDB1-FLAG or FLAG-CUL4 protein in the input, the anti-FLAG immunoprecipitate (IP) and the anti-DIM-5 IP fractions. In contrast, neither FLAG-tagged protein nor DIM-5 was detected in the mock IP (Figure 3C). Similarly, we performed CoIP experiments using a strain expressing 3XFLAG-tagged DIM-9 (DIM-9-FLAG) and 3XHA-tagged DIM-5 (DIM-5-HA). Western blots revealed both proteins in the input, the anti-FLAG IP, and the anti-HA IP fractions, confirming that these proteins interact *in vivo* (Figure 3D).

Our finding that the products of *dim-7*, *dim-8*, *dim-9* and *cul4* genes co-purified with DIM-5 and are all absolutely required for DIM-5 function, and our confirmation of key interactions by CoIP experiments, led us to conclude that DIM-5 is part of a complex necessary for DNA methylation in Neurospora. We will refer to this complex as DCDC (the DIM-5/-7/-9, CUL4/DDB1 complex). We were interested to learn whether some or all of the identified DCDC proteins would co-purify with CUL4. To investigate this, we engineered a strain expressing CUL4 fused to a tandem HAT-FLAT affinity tag [31], purified the tagged protein, and identified associated proteins by mass spectrometry. We identified peptides corresponding to CUL4 (49% coverage), DDB1 (44% coverage), DIM-7 (30% coverage) and DIM-9 (28% coverage). Interestingly, DIM-5, NFH-1 and NFH-2 were not identified in the purified fraction (Table S2), suggesting that DIM-5 only associates with a fraction of the total CUL4/DDB1 protein complex in the cell, consistent with the expectation that CUL4/DDB1 serves as a scaffold for more than one complex.

Purification of CUL4-associated proteins revealed additional proteins that do not seem to be members of DCDC, but are known to interact with CUL4/DDB1 in other organisms [32], [33] (Table S2). These include several WD40 domain-containing proteins, which presumably correspond to Neurospora DCAFs, plus members of the COP9 signalosome complex. Cullin proteins are typically modified post-translationally by attachment of the small ubiquitin-like protein, NEDD8. We identified peptides corresponding to Neurospora NEDD8 in the band that contained CUL4, suggesting that Neurospora CUL4 is neddylated. We examined DNA methylation levels in mutant strains lacking individual DCAFs or components of the COP9 signalosome complex and found normal DNA methylation in these strains (Table S2). These data suggest that Neurospora CUL4 and DDB1 interact with DCAFs to form distinct complexes that participate in various cellular processes.

### DCDC is required for normal chromosome segregation

**Figure 4:**
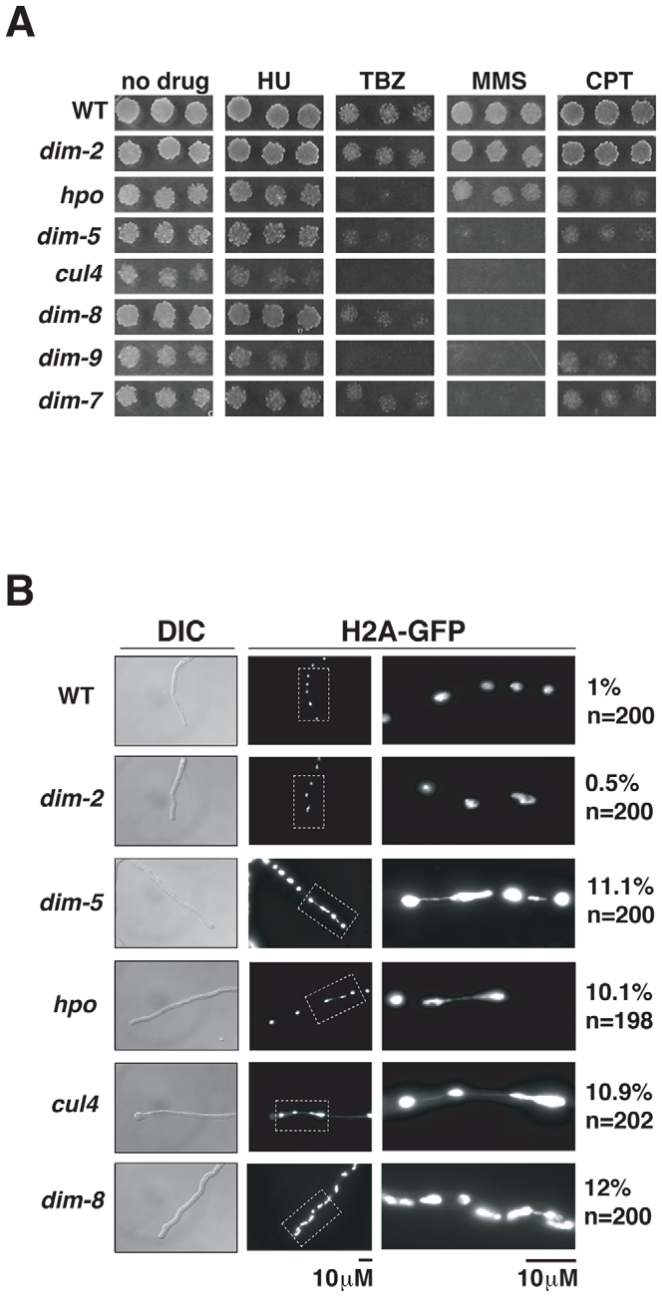
Heterochromatin is required for normal chromosome segregation. (A) Suspensions of 10^4^, 10^3^ or 10^2^ conidia of the indicated strains were spot-tested on media with or without hydroxyurea (HU), methyl methanesulfonate (MMS), camptothecin (CPT) or thiabendazole (TBZ). (B) The distribution of H2A-GFP in growing hyphal tips in wildtype, *dim-2*, *dim-5*, *dim-8 and cul4* strains. The frequency of observed chromatin bridges/total number of nuclei is shown beside each micrograph.

Mutant strains lacking components of DCDC exhibit growth defects (representative data shown for *cul4* in Figure S3), similar to previously reported defects observed for *dim-5* and *hpo* strains [16], [17]. To test heterochromatin-deficient mutants for specific defects in transcription, centromere function, and DNA repair, we tested their sensitivity to diagnostic drugs. Serial dilutions of conidia of wildtype, *dim-2*, *hpo* and DCDC-defective strains were spot-tested on unsupplemented medium and media supplemented with hydroxyurea (HU; ribonucleotide reductase inhibitor), methyl methanesulfonate (MMS; alkylating agent), camptothecin (CPT; topoisomerase I inhibitor) or thiabendazole (TBZ; microtubule inhibitor). All strains were able to grow on HU (Figure 4A). In contrast, *hpo* and the DCDC mutants were hypersensitive to TBZ, whereas the *dim-2* and wildtype control strains were not. Interestingly, the DCDC mutants, but not *hpo*, were hypersensitive to MMS, suggesting that some functions of H3K9me3 are not dependent on HP1. Finally, *cul4* and *dim-8* mutants were hypersensitive to CPT, whereas all other strains tested grew on this drug, consistent with a role for CUL4/DDB1 in additional cellular processes, presumably mediated by additional DCAFs.

The sensitivity to the microtubule inhibitor TBZ observed for *hpo* and DCDC mutants suggests H3K9me3 and HP1 are important for centromere function. To test this possibility, we examined chromosome segregation in live cells using a GFP-tagged H2A to visualize chromatin. Indeed, *hpo*, *dim-5*, *cul4* and *dim-8* mutants displayed high frequencies of lagging chromosomes, indicating that centromere function is impaired in these strains. All of these mutants showed chromosome bridges associated with approximately 10% of the nuclei, whereas evidence of lagging chromosomes was rarely observed in wildtype or *dim-2* stains (Figure 4B).

### DIM-7 recruits DIM-5 to form DCDC

**Figure 5:**
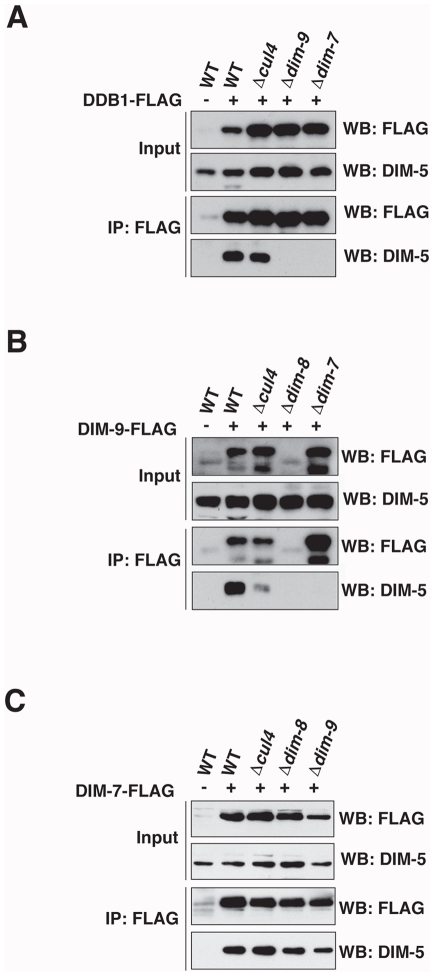
DIM-7 recruits DIM-5 to form DCDC. Immunoprecipitation experiments were performed using extracts from the indicated wildtype, *cul4*, *dim-8*, *dim-9* or *dim-7* strains with or without (A) DDB1-FLAG, (B) DIM-9-FLAG or (C) DIM-7-FLAG (indicated by + or −). The input fraction and the α-FLAG immunoprecipitate (IP:α-FLAG) were subjected to western blotting and probed with antibodies to α-FLAG or α-DIM-5 as indicated (WB).

In an attempt to determine which components of DCDC are responsible for recruiting DIM-5 to the complex, we initially tested for direct interaction between DIM-5 and each DCDC member by the yeast two-hybrid assay but these experiments failed to demonstrate a direct interaction between DIM-5 and any other DCDC component (data not shown). We therefore carried out CoIP experiments to test individual DCDC knockout strains for their ability to support pair-wise interactions between DIM-5 and other members of DCDC. FLAG-tagged versions of DDB1, DIM-9 and DIM-7 were expressed from their native loci. Tagged proteins were precipitated with anti-FLAG antibodies, and the input and IP fractions were interrogated with anti-FLAG and anti-DIM-5 antibodies. Immunoprecipitation of DDB1-FLAG, DIM-9-FLAG and DIM-7-FLAG revealed that all three proteins interact with DIM-5 in both wildtype and *cul4* strains (Figure 5A–5C), indicating that CUL4 is dispensable for interaction of DIM-5 with other DCDC components. We note that although the DIM-9-DIM-5 interaction appears reduced in the experiment illustrated (Figure 5B), we observed increased interaction between these two proteins in a replicate experiment (Figure S4). Interestingly, immunoprecipitation of DDB1-FLAG failed to reveal a DDB1-DIM-5 interaction in the *dim-9* or *dim-7* strains (Figure 5A), suggesting that DIM-9 and DIM-7 mediate the indirect interaction of DIM-5 with DDB1.

Western blots of both the input and IP fractions revealed that DIM-9-FLAG levels were markedly reduced in the *dim-8* strain (Figure 5B), suggesting that DIM-9 stability depends on DDB1. Consistent with this, DIM-5 was not found in the DIM-9-FLAG IP fraction from the *dim-8* strain (Figure 5B). Yeast two-hybrid assays revealed an interaction between DIM-9 and DDB1 (data not shown), suggesting that these proteins interact directly, as expected. Together, these data suggest that direct interaction of DDB1 and DIM-9 is important for DIM-9 stability.

DIM-9-FLAG was readily detectable in the *dim-7* strain, but DIM-5 was not found in the DIM-9-FLAG IP fraction of this strain (Figure 5B). These findings indicate that the DIM-9-DIM-5 interaction depends on DIM-7. In contrast to the situation for DDB1 and DIM-9, the interaction of DIM-5 and DIM-7-FLAG was independent of all other DCDC members. Indeed, DIM-5 was detected in the IP fraction following immunoprecipitation of DIM-7-FLAG from wildtype, *cul4*, *dim-8*, and *dim-9* strains (Figure 5C). These data suggest that DIM-7 is required to mediate interaction of DIM-5 with DCDC, most likely *via* DIM-9.

### DIM-7 directs DIM-5 to heterochromatin domains

**Figure 6:**
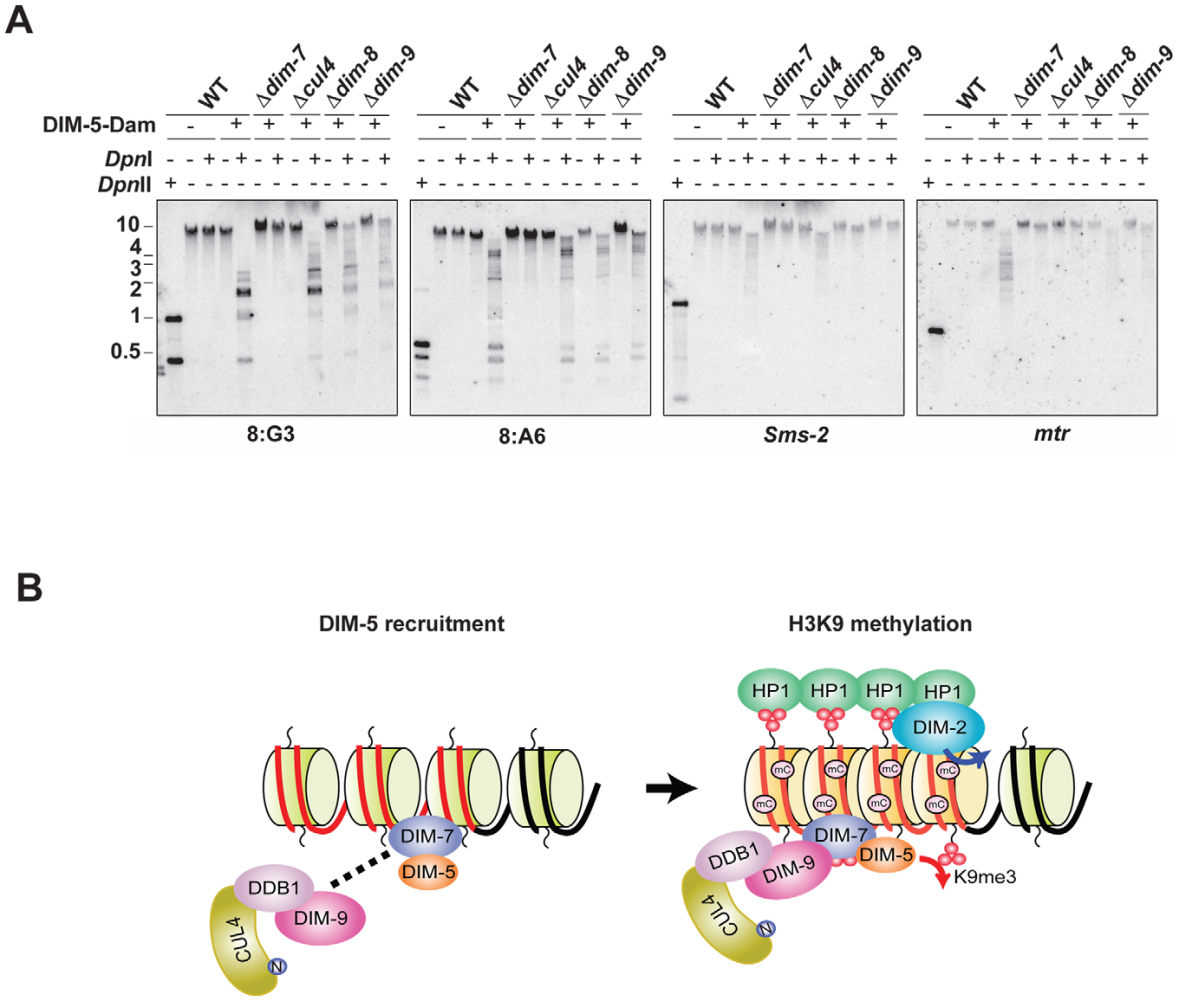
DIM-7, but not CUL4/DDB1^DIM-9^, is required for recruitment of DIM-5 to heterochromatin domains. (A) Genomic DNA from wildtype, which does not express DIM-5-Dam, as well as Dim+, *dim-7*, *dim-8*, *cul4* and *dim-9* strains expressing DIM-5-Dam were incubated with or without *Dpn*I, which cuts GATC only when the adenine is methylated. As an indicator of completely digested DNA, genomic DNA from the wildtype strain was incubated with the cytosine methylation-insensitive enzyme *Dpn*II. Digested DNA was used for Southern hybridizations with probes corresponding to the indicated heterochromatic 8:A6 and 8:G3 regions and the euchromatic *mtr* and *Sms-2* genes. (B) A model for H3K9 methylation by DCDC is shown (see text).

We recently adapted the DamID technique [18], [39] to test for chromatin association of DIM-5 and showed that DIM-7 is required to target DIM-5 to heterochromatin domains. Because DIM-7 is required to recruit DIM-5 to form the DCDC, we tested if the other components of DCDC are also required for association of DIM-5 with chromatin regions destined to be methylated. We introduced a DIM-5-Dam fusion construct into the *cul4*, *dim-8* and *dim-9* strains. We then tested for Dam activity in these strains, as well as positive- (wildtype) and negative- (*dim-7*) control strains by treating genomic DNA with *Dpn*I, which specifically cuts GATC sites containing methylated adenines, but does not digest unmethylated GATC sites (Figure 6A). The digested DNA was fractionated by electrophoresis and probed for the heterochromatin regions 8:G3 and 8:A6, as well as for the euchromatic genes *mtr* and *Sms-2*. For the wildtype, *cul4*, *dim-8* and *dim-9* strains, the heterochromatin probes detected low molecular weight fragments corresponding to completely digested DNA and some intermediate molecular weight fragments corresponding to partially digested DNA. In contrast, only high molecular weight DNA was detected in the *dim-7* background. Importantly, probes for *Sms-2* and *mtr* hybridized to high molecular weight DNA corresponding to largely undigested DNA in all strains. These data suggest that DIM-7 is required to recruit DIM-5 to heterochromatin domains, while the remaining DCDC members are not.

## Discussion

DNA methylation, which is frequently associated with heterochromatin, is essential for development, genome defense, genome imprinting and X-chromosome inactivation [1]–[4], and misregulation of DNA methylation has been implicated in disease [8]. Unfortunately, the mechanisms that direct heterochromatin and DNA methylation are not well understood. To uncover the mechanisms responsible for regulating DNA methylation in Neurospora, we carried out three independent lines of investigation: 1) We selected for mutants that are defective in DNA methylation; 2) we identified DIM-5-associated proteins by mass spectrometry; and 3) we made, and tested the effects of, mutations in candidate genes, such as *cul4*. These approaches proved complementary, revealing that a complex of DIM-5, DIM-7, CUL4, DDB1 and DIM-9, which we named DCDC, is required for H3K9 methylation and DNA methylation.

CUL4 and DDB1 are conserved from *S. pombe* to humans and are known to participate in a variety of cellular processes [32], [33]. Our discovery that CUL4 and DDB1 are required for DNA methylation is consistent with a report published while this paper was in preparation [40]. Our more comprehensive analyses revealed additional components of a DIM-5-containing complex and a hierarchy of interactions within the complex. Distinct functions of CUL4/DDB1 complexes are mediated by variable, WD40 domain-containing subunits called DCAFs, which interact directly with DDB1 and are thought to determine the substrate specificities of the various CUL4/DDB1 ubiquitin ligase complexes. DIM-9 is a WD40 domain-containing protein, suggesting that DIM-9 is the DCAF component of DCDC. Consistent with this, yeast two-hybrid analyses revealed that DIM-9 interacts directly with DDB1. In addition, DDB1 is required for stability of DIM-9. Our data also indicate that DIM-9 is required to mediate interaction of DIM-7/DIM-5 with CUL4/DDB1. These findings would be consistent with the possibility that DIM-7 or DIM-5 is a substrate for the CUL4/DDB1^DIM-9^ ubiquitin ligase; however, several attempts to identify ubiquitylated forms of either protein in Neurospora extracts were unsuccessful (data not shown). In addition, we note that sequence alignments of fungal DIM-7 homologues reveal only a handful of conserved residues, none of which are lysine residues [18], suggesting that ubiquitylation of DIM-7 is unlikely.

Although the putative substrate of CUL4/DDB1^DIM-9^ is unknown, our results are consistent with the possibility that the complex does serve as a ubiquitin ligase. We found that DIM-5 recruitment to heterochromatin domains is independent of CUL4, DDB1 and DIM-9. These data demonstrate that recruitment of DIM-5 to heterochromatin is not sufficient to direct H3K9 methylation. Similarly, recent work in *S. pombe* demonstrated that tethering of the Clr4 KMT to chromatin is not sufficient to direct H3K9 methylation in the absence of Rik1 [41]. It is notable that recombinant DIM-5 shows robust and specific methyltransferase activity on naked histones but not on nucleosomal substrates [22]. One possible role for the CUL4/DDB1^DIM-9^ components of DCDC would be to direct ubiquitination of a histone, thereby making H3 more accessible for methylation by DIM-5.

Interestingly, purification of CUL4/DDB1 complexes from mammalian cells has uncovered several DCAFs that are also components of histone lysine methyltransferase complexes [32], [33]. Furthermore, knock down of *cul4* or *dim-*8 (DDB1) gene expression led to reduced methylation at several histone residues [30], consistent with a general role for CUL4 and DDB1 in histone methylation. Here we observed normal levels of H3K4, H3K27, H3K36, H3K79 and H4K20 methylation in CUL4- and DDB1-deficent strains, indicating that these proteins are not required for general histone methylation in Neurospora. Rather, they specifically regulate H3K9 methylation. CUL4- and DDB1-deficient strains exhibited hypersensitivity to the topoisomerase I inhibitor, CPT, whereas mutants deficient in other members of DCDC did not, supporting the expectation that CUL4 and DDB1 perform functions in addition to their function required for heterochromatin formation. Consistent with this, purification of CUL4 revealed additional DCAF proteins, suggesting that CUL4 and DDB1 form multiple ubiquitin ligase complexes as in other organisms.

We observed that DCDC and HP1 mutants are hypersensitive to the microtubule inhibitor TBZ, and that these strains exhibit high frequencies of lagging chromosomes. These data suggest that H3K9 methylation and HP1 are important for chromosome segregation in Neurospora, similar to the case in mammals, Drosophila and *S. pombe* [42]–[45]. This observation provides an explanation for the poor growth of Neurospora heterochromatin-deficient strains [16], [17].

Although *S. pombe* lacks DNA methylation, CLRC, a Clr4-containing complex that is essential for H3K9 methylation in this yeast [28], [29], resembles *N. crassa* DCDC. These complexes exhibit several significant differences, however. First, DCDC includes the conserved CUL4 binding partner DDB1, whereas CLRC utilizes the DDB1-like protein Rik1. In addition, these subunits appear to perform different functions. Rik1 is essential for RNAi-dependent recruitment of CLRC to heterochromatin nucleation sites [46], while DIM-5 recruitment to heterochromatin domains is independent of DDB1. DIM-5 recruitment is also independent of the DCDC components CUL4 and DIM-9, whereas recruitment of *S. pombe* Clr4 to heterochromatin is dramatically reduced in a Cul4 mutant strain [29], [46]. In contrast, DIM-7 is required to target DIM-5 to heterochromatin domains [18]. Another distinction between *S. pombe* CLRC and Neurospora DCDC involves the requirement of a 14-3-3 domain-containing subunit. In *S. pombe*, Rad24 co-purified with CLRC and is required for heterochromatic gene silencing and siRNA production [36]. Purification of Neurospora DIM-5 revealed NFH-1 and -2, but inactivation of the corresponding genes did not markedly effect DNA methylation, indicating that these proteins are not essential for maintenance of heterochromatin in Neurospora. These differences between *S. pombe* CLRC and Neurospora DCDC are not surprising given that these fungi employ different mechanisms to regulate heterochromatin formation. Indeed, work with *S. pombe* revealed that CLRC interacts with the Argonaute-containing RITS complex via the protein Stc1 to target H3K9 methylation [46], [47], whereas in Neurospora, H3K9 and DNA methylation do not depend on RNAi, but instead are directed by A:T-rich DNA [9], [13], [14], [48].

Mass spectrometry of DIM-5-associated proteins revealed that DIM-7 was the best represented DIM-5-associated component of DCDC, suggesting that DIM-5 and DIM-7 may interact directly. Consistent with this possibility, we demonstrated that the DIM-5/DIM-7 interaction is independent of other DCDC components, whereas DIM-7 is required for interaction of DIM-5 with DDB1 and DIM-9. Taken together, these data suggest that DIM-7 is required to recruit DIM-5 to form DCDC and lead us to propose a model (Figure 6B) in which DIM-5 and DIM-7 directly interact. We propose a two-step mechanism for H3K9 methylation by DCDC. First, DIM-7 recruits DIM-5 to form DCDC and somehow targets the complex to A:T-rich relics of RIP, by either a direct or indirect interaction with chromatin. We found that histones H3, H2A and H2B co-purify with DIM-7 (unpublished data of Z. Lewis and E. Selker), lending support to this model. In the second step, DIM-5 performs *tri*-methylation of H3K9 associated with RIP'd DNA in a CUL4/DDB1^DIM-9^-dependent manner. DIM-7 is not well conserved, but it appears to be a distant homolog of the CLRC component Raf-2. Therefore it would be interesting to know if Raf2 is responsible for recruitment of Clr4 to form the CLRC complex.

It seems quite possible that H3K9 KMTs exist in multi-protein complexes, generally [49], and that KMT-interacting proteins are important for targeting H3K9 methylation to appropriate chromatin domains. Purification of the mammalian H3K9 KMTs, Suv39H1, Suv39H2, G9a and SETDB1, did not reveal an interaction with CUL4 or DDB1 proteins [49] but these results do not rule out a possible role for a mammalian CUL4/DDB1 complex in heterochromatin formation. Moreover, a weak but biologically relevant interaction between mammalian H3K9 KMTs and CUL4/DDB1 proteins could be missed in analyses of affinity-purified proteins. Interestingly, mammalian cells in which DDB1 and CUL4 expression were knocked down showed reduced levels of H3K9 methylation [30], suggesting that these proteins may play a conserved role in heterochromatin formation from fungi to mammals.

## Methods

### Neurospora growth and molecular analyses

All strains used in this study are listed in Table S3. *N. crassa* strains were maintained, grown and crossed using previously described procedures [50]. Neurospora transformation [51], DNA isolation [52], Southern blotting [13], isolation of nuclei [53], fluorescence microscopy [17], protein isolation, histone isolation, coimmunoprecipitation [23] and construction of FLAG-tagged strains [31] were performed as described. All primers used in this study are listed in . Detailed descriptions of knock-out and epitope-tagged strain construction and a list of antibodies used for western blot analyses and coimmunoprecipitation experiments are available in the supplementary information.

In Neurospora, transforming DNA is typically integrated into the genome in an apparently random manner [38]. We therefore performed approximately three hundred transformations of our methylation reporter strain (N2977) as an attempt to generate mutations associated with the introduced DNA and selected for basta-resistant transformants as described in Text S1.

### Identification of DIM-5–associated proteins

Construction of HAT-FLAG-tandem-affinity-tagged strains and the two-step purification were performed as described (Honda and Selker, 2009). Purified samples were separated by SDS-PAGE. As expected, DIM-5 was resolved with an apparent molecular weight of 38 kD. Gel slices containing bands were excised, washed and in-gel digested with trypsin overnight at 37°C. Tryptic peptides were separated by nano-HPLC (Rheos 2000) coupled to a 3D-ion trap mass spectrometer (LCQ Deca XP, both Thermo Fisher Scientific). The LC system was equipped with a capillary column with an integrated nanospray tip (100 µm i.d. ×100 mm, Swiss BioAnalytics AG) filled with Magic C18 (Michrom Bioresources, Inc.). Samples were loaded on a Peptide CapTrap (Michrom BioResources, Inc.) using a CTC PAL autosampler (CTC Analytics AG). Elution was performed with a gradient of 0 – 45% solvent B in 30 min at a flow rate of 500 nL/min. Solvent A consisted of 0.1% formic acid/2% acetonitrile; solvent B was composed of 0.1% formic acid/80% acetonitrile. In the data-dependent mode, the mass spectrometer cycled through four analyses, one MS full scan followed by MSMS scans for each of the three most intense peaks. Peptides were identified searching UniProt 15.14 using Mascot Distiller 2.3 for data extraction and conversion and Mascot 2.2 (Matrix Science). Results were compiled with Scaffold 2.06.

### Phenotypic analyses of heterochromatin mutants

For drug sensitivity assays, serial dilutions of conidia were spot-tested on media with or without HU (8 mM), MMS (0.015%), CPT (0.3 µg/ml), or TBZ (0.5 µg/ml) obtained from Sigma Aldrich. To facilitate tracking chromatin cytologically, H2A-GFP (see Text S1) in growing hyphae was visualized using a Zeiss Axioplan 2 Imaging system with 100X oil immersion lens. Bright field and fluorescence images were collected using Images and processed using Axiovision (4.6.3) and Adobe Photoshop CS (version 8) software. Approximately 200 hyphal tips were counted for each culture and the number of tips that displayed nuclei with lagging chromosome bridges was noted to quantify the chromosome segregation defects.

## Supporting Information

Figure S1 CUL4 is essential for DNA methylation in Neurospora. Southern hybridizations were performed using genomic DNA from the indicated strains digested with the cytosine-methylation-sensitive BfuCI (B) and -insenstive DpnII (D) endonucleases. The blots were probed for genomic regions (indicated below each blot) that are normally methylated in wildtype. EtBr refers to the ethidium bromide stain.
(0.12 MB PDF)

Figure S2 Mutation of NCU01656, a gene encoding a candidate DIM-5-associated protein, is responsible for loss of methylation in *dim-9* strains. Southern hybridizations were performed using genomic DNA from wildtype, a *dim-9* strain obtained in our mutant hunt, and a *dim-9* strain transformed with a wildtype copy of NCU01656. DNA was digested with BamHI and EcoRI endonucleases. The blot was probed for 8:A6, a region that is normally methylated. Methylation of an EcoRI site produces a slower migrating band, indicated by an asterisk.
(0.54 MB PDF)

Figure S3 *cul4* mutants exhibit growth defects. (A) Cultures of wildtype and *cul4* mutant strains after 7 days of growth at 32°C. *cul4* mutant exhibits slow growth and reduced conidiation. (B) Sibling *cul4* mutant progeny from a cross of wildtype and *cul4* are shown. (C) The linear growth rate (mm/hour) of four wildtype progeny (blue) and four *cul4* siblings (red).
(5.27 MB PDF)

Figure S4 Interaction between DIM-9 and DIM-5 does not depend on CUL4. Immunoprecipitation experiments were performed using extracts from the indicated wildtype, *cul4*, *dim-8*, or *dim-7* strains with DIM-9-FLAG. The input fraction and the α-FLAG immunoprecipitate (IP: α-FLAG) were subjected to western blotting and probed with antibodies to α-FLAG or α-DIM-5 as indicated (WB). The experiment shown is an independent biological replicate of the experiment shown in Figure 5B.
(5.71 MB PDF)

Table S1 DIM-5-associated proteins.
(0.14 MB DOCX)

Table S2 CUL4-associated proteins.
(0.11 MB DOCX)

Table S3 Strains used in this study.
(0.13 MB DOCX)

Table S4 Oligos used in this study.
(0.13 MB DOCX)

Text S1 Supplemental experimental procedures.
(0.11 MB DOCX)
