# Supplementary figures and images for: DNA Methylation and Normal Chromosome Behavior in Neurospora Depend on Five Components of a Histone Methyltransferase Complex, DCDC
Source: PLoS Genet. 2010 Nov 4;6(11):e1001196. doi: 10.1371/journal.pgen.1001196 (PMC2973830; doi:10.1371/journal.pgen.1001196)

**A**

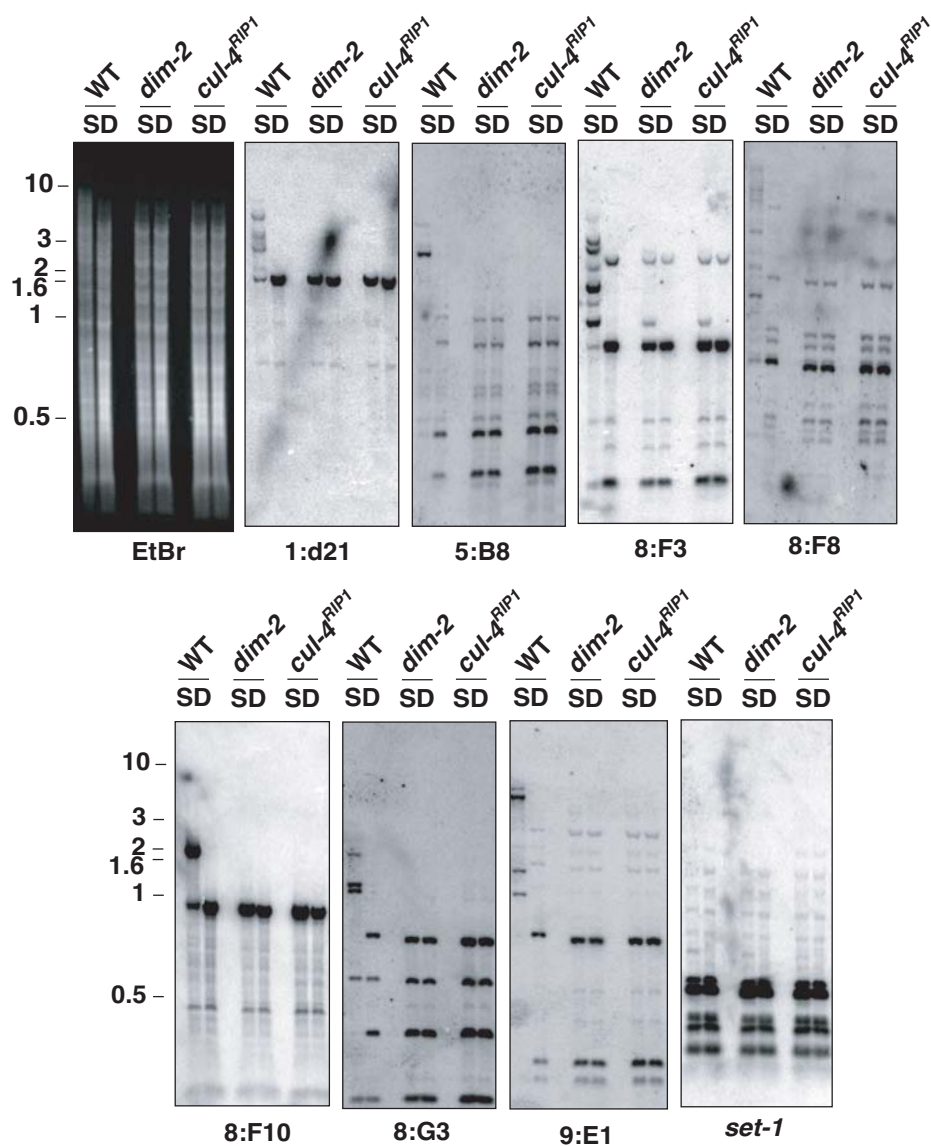

**B**

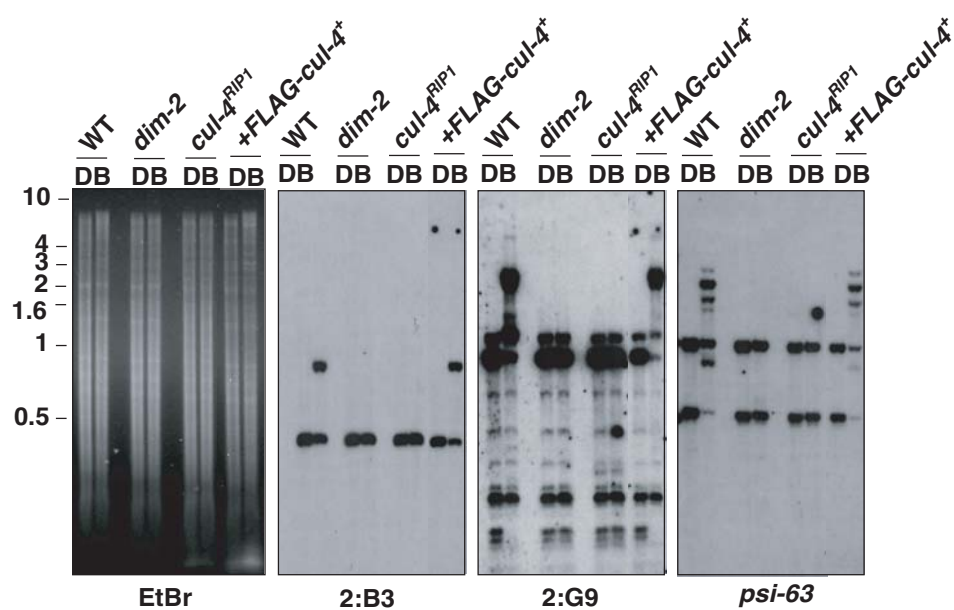

Supplement: Figure S1 — CUL4 is essential for DNA methylation in Neurospora. Southern hybridizations were performed using genomic DNA from the indicated strains digested with the cytosine-methylation-sensitive BfuCI (B) and -insenstive DpnII (D) endonucleases. The blots were probed for genomic regions (indicated below each blot) that are normally methylated in wildtype. EtBr refers to the ethidium bromide stain. (0.12 MB PDF) [file pgen.1001196.s001.pdf]

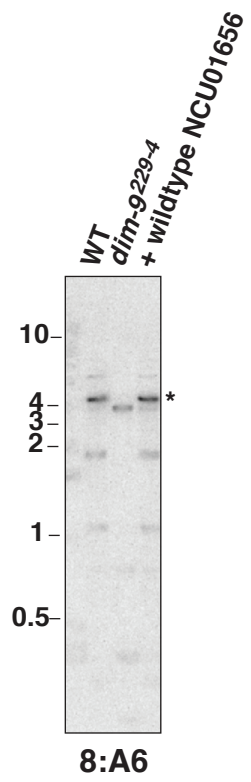

Supplement: Figure S2 — Mutation of NCU01656, a gene encoding a candidate DIM-5-associated protein, is responsible for loss of methylation in dim-9 strains. Southern hybridizations were performed using genomic DNA from wildtype, a dim-9 strain obtained in our mutant hunt, and a dim-9 strain transformed with a wildtype copy of NCU01656. DNA was digested with BamHI and EcoRI endonucleases. The blot was probed for 8:A6, a region that is normally methylated. Methylation of an EcoRI site produces a slower migrating band, indicated by an asterisk. (0.54 MB PDF) [file pgen.1001196.s002.pdf]

**A**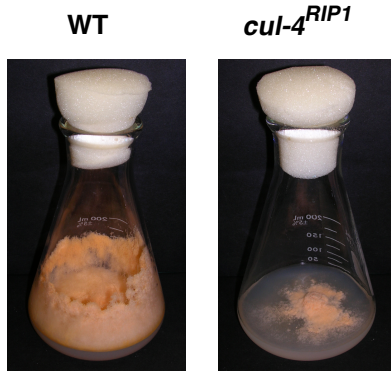**B**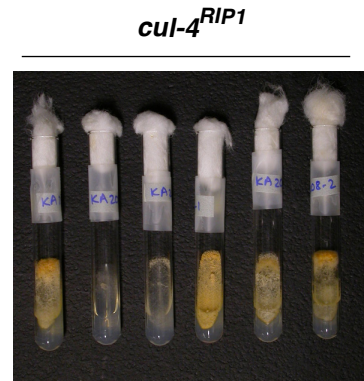**C**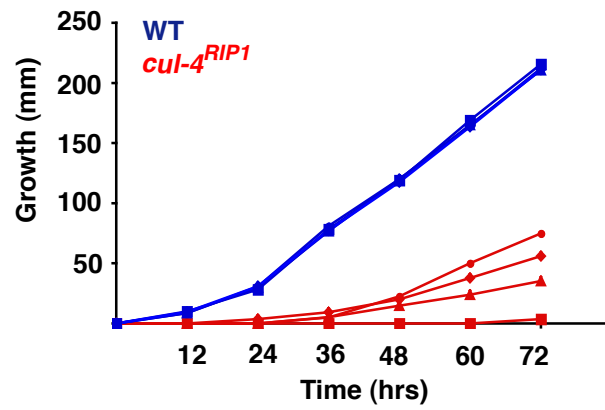

Supplement: Figure S3 — cul4 mutants exhibit growth defects. (A) Cultures of wildtype and cul4 mutant strains after 7 days of growth at 32°C. cul4 mutant exhibits slow growth and reduced conidiation. (B) Sibling cul4 mutant progeny from a cross of wildtype and cul4 are shown. (C) The linear growth rate (mm/hour) of four wildtype progeny (blue) and four cul4 siblings (red). (5.27 MB PDF) [file pgen.1001196.s003.pdf]

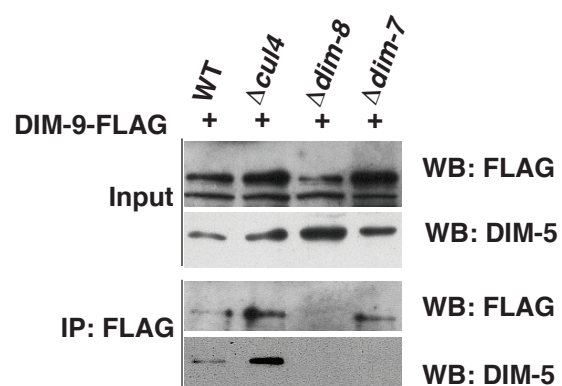

Supplement: Figure S4 — Interaction between DIM-9 and DIM-5 does not depend on CUL4. Immunoprecipitation experiments were performed using extracts from the indicated wildtype, cul4, dim-8, or dim-7 strains with DIM-9-FLAG. The input fraction and the α-FLAG immunoprecipitate (IP: α-FLAG) were subjected to western blotting and probed with antibodies to α-FLAG or α-DIM-5 as indicated (WB). The experiment shown is an independent biological replicate of the experiment shown in Figure 5B. (5.71 MB PDF) [file pgen.1001196.s004.pdf]
