# Supplementary material for: DNA Methylation and Normal Chromosome Behavior in Neurospora Depend on Five Components of a Histone Methyltransferase Complex, DCDC
Source: PLoS Genet. 2010 Nov 4;6(11):e1001196. doi: 10.1371/journal.pgen.1001196 (PMC2973830; doi:10.1371/journal.pgen.1001196)
Supplement: Table S1 — DIM-5-associated proteins. (0.14 MB DOCX) [file pgen.1001196.s005.docx]

**Table S1. DIM-5-associated proteins**

| **Gene #^1^** | **Predicted Protein^2^** | **MW**  **(kDa) ^3^** | **Number of unique peptides^4^** | **Protein coverage^5^** |
| --- | --- | --- | --- | --- |
| NCU02744 | 60S ribosomal protein L9 | 22 | 11 | 44% |
| NCU02295 | Phosphatidylinositol-4-phosphate 5-kinase its3 | 103 | 26 | 38% |
| NCU08334 | Predicted protein | 124 | 34 | 31% |
| NCU01728 | Related to 6-phosphofructo-2-kinase | 90 | 27 | 31% |
| **NCU04152** | **DIM-7** | **74** | **13** | **25%** |
| **NCU04402** | **DIM-5** | **38** | **10** | **25%** |
| NCU01511 | Probable Ni-binding urease accessory protein (UreG) | 31 | 5 | 24% |
| NCU09212 | Putative uncharacterized protein | 69 | 10 | 21% |
| NCU07829 | 60S ribosomal protein L7 | 29 | 3 | 19% |
| NCU00294 | 60S ribosomal protein L10a | 24 | 3 | 19% |
| **NCU03300** | **NFH-1** | **16** | **2** | **19%** |
| NCU02797 | UTP-glucose-1-phosphate uridylyltransferase | 58 | 6 | 15% |
| NCU05226 | Putative uncharacterized protein | 76 | 6 | 14% |
| NCU09693 | Putative uncharacterized protein | 35 | 2 | 14% |
| **NCU06605** | **DDB1** | **129** | **12** | **13%** |
| **NCU02806** | **NFH-2** | **29** | **3** | **12%** |
| NCU06431 | 40S ribosomal protein S22 | 15 | 1 | 12% |
| **NCU00272** | **CUL4** | **113** | **8** | **11%** |
| NCU09602 | Heat shock 70 protein | 71 | 7 | 11% |
| NCU02957 | Putative uncharacterized protein | 53 | 3 | 10% |
| NCU09237 | Related to hnRNP protein E2 | 51 | 3 | 9% |
| NCU06226 | 60S ribosomal protein L25 | 17 | 1 | 9% |
| NCU00043 | Serine/threonine-protein phosphatase PP1 | 36 | 2 | 9% |
| NCU08991 | (Q8NK13) Negative regulator sulfur controller-3 | 20 | 1 | 9% |
| **NCU01656** | **DIM-9** | **137** | **8** | **8%** |
| NCU01747 | Putative uncharacterized protein | 138 | 11 | 8% |
| NCU00541 | Putative uncharacterized protein | 44 | 2 | 8% |
| NCU03860 | (Putative uncharacterized protein | 17 | 1 | 8% |
| NCU07826 | 40S ribosomal protein S19 | 17 | 1 | 7% |
| NCU05274 | Eukaryotic translation initiation factor 5A | 18 | 1 | 7% |
| NCU09861 | Putative uncharacterized protein | 73 | 3 | 7% |
| NCU00951 | Inorganic pyrophosphatase | 33 | 1 | 7% |
| NCU02003 | Elongation factor 1-alpha | 50 | 4 | 6% |
| NCU03500 | Putative uncharacterized protein | 58 | 2 | 6% |
| NCU02542 | Hexokinase | 54 | 2 | 5% |
| NCU02274 | Serine hydroxymethyltransferase, cytosolic | 53 | 2 | 5% |
| NCU08167 | Putative uncharacterized protein | 60 | 2 | 5% |
| NCU11129 | Putative uncharacterized protein | 69 | 2 | 5% |
| NCU04173 | Actin | 42 | 1 | 4% |
| NCU01881 | Putative uncharacterized protein | 80 | 2 | 4% |
| NCU05285 | Putative uncharacterized protein | 48 | 1 | 4% |
| NCU06110 | CyPBP37 protein | 37 | 1 | 4% |
| NCU09670 | Putative uncharacterized protein | 203 | 4 | 3% |
| NCU06803 | Glutamate decarboxylase | 59 | 1 | 3% |
| NCU02193 | Pyruvate decarboxylase | 62 | 1 | 3% |
| NCU02075 | Heat shock protein SSB1 | 66 | 1 | 2% |
| NCU04865 | Putative uncharacterized protein | 281 | 2 | 1% |

^1^ The gene identification #’s were obtained from the Neurospora crassa genome database (http://www.broadinstitute.org/annotation/genome/neurospora/MultiHome.html). The rows highlighted in bold indicate members of the DCDC complex characterized in the present manuscript.

^2^ The protein descriptions were obtained from the Uniprot database (http://www.uniprot.org)

^3^ Molecular weight is shown in kilodaltons.

^4^ The # of unique peptides identified by mass spectrometry was determined using Scaffold 2.06 with the minimum peptide probability set to 95%

^5^ The % coverage of each protein by peptides identified by mass spectrometry was determined using Scaffold 2.06 with the minimum peptide probability set to 95%.
