# Supplementary material for: DNA Methylation and Normal Chromosome Behavior in Neurospora Depend on Five Components of a Histone Methyltransferase Complex, DCDC
Source: PLoS Genet. 2010 Nov 4;6(11):e1001196. doi: 10.1371/journal.pgen.1001196 (PMC2973830; doi:10.1371/journal.pgen.1001196)
Supplement: Table S2 — CUL4-associated proteins. (0.11 MB DOCX) [file pgen.1001196.s006.docx]

**Table S2. CUL4-associated proteins**

| **Gene #^1^** | **Predicted Protein^2^** | **MW^3^ (kDa)** | **Number of unique peptides^4^** | **Percent coverage^5^** | **DNA methylation^6^** |
| --- | --- | --- | --- | --- | --- |
| **NCU00272** | **CUL-4** | **113** | **42** | **43** | **Absent** |
| **NCU06605** | **DDB-1** | **129** | **41** | **38** | **Absent** |
| NCU05271 | **Predicted protein** | 147 | 37 | 36 | Normal |
| NCU09730 | Kinesin | 102 | 30 | 34 | ND |
| NCU08334 |  | 124 | 28 | 31 | ND |
| **NCU01656** | **DIM-9** | **137** | **24** | **26** | **Absent** |
| NCU01728 | Phosphofructokinase | 71 | 19 | 41 | ND |
| NCU02295 | Phosphotidyl-inositol-4 phosphate-5 kinase | 103 | 18 | 24 | ND |
| NCU02151 | WD-40 protein LEC 18B | 76 | 16 | 25 | Normal |
| NCU09325 | WD-40 protein, Het e | 103 | 15 | 17 | Normal |
| **NCU04152** | **DIM-7** | **74** | **15** | **30** | **Absent** |
| NCU02193 | Pyruvate carboxylase | 62 | 13 | 31 | ND |
| NCU07019 | CSN-6 | 54 | 14 | 33 | Normal |
| NCU07670 | CYT 19 ATP helicase | 69 | 14 | 25 | ND |
| NCU07361 | CSN-4 | 48 | 9 | 33 | Normal |
| NCU02003 | eEFI alpha | 50 | 8 | 19 | Essential gene; normal in heterokaryon |
| NCU05226 | ABC transporter | 76 | 8 | 15 | ND |
| NCU11350 | Glucosamine | 77 | 7 | 14 | ND |
| NCU01881 | Peptidyl prolyl cis trans isomerase | 94 | 7 | 12 | Normal |
| NCU01797 | NRC-2 kinase | 69 | 7 | 12 | ND |
| NCU01408 | CSN-3 | 55 | 6 | 13 | Normal |
| NCU00157 | CSN-1 | 48 | 6 | 19 | Normal |
| NCU00467 | CSN-5 | 37 | 6 | 20 | Normal |
| NCU02744 | 60S ribosomal protein | 22 | 6 | 24 | ND |
| NCU02075 | HSP-70 | 66 | 6 | 12 | ND |
| NCU03668 | WD-40 protein, lethal denticleless | 85 | 6 | 6.7 | Essential gene; normal in heterokaryon |
| NCU08342 | CSN-7 | 45 | 6 | 17 | Normal |
| NCU09602 | HSP-70-1 | 71 | 5 | 8.7 | ND |
| NCU07700 | COT-3 kinase | 93 | 5 | 5.2 | ND |
| NCU00593 | CSN-2 | 57 | 5 | 15 | Normal |
| NCU04173 | Actin | 42 | 4 | 9.3 | ND |
| NCU03500 | aminotransferase | 58 | 4 | 6.4 | ND |
| NCU03309 | NEDD-8 | 9 | 3 | 51 | Essential gene; normal in heterokaryon |
| NCU03200 | SCK-1 kinase | 100 | 3 | 4.8 | ND |
| NCU02356 | WC-1 | 126 | 2 | 2.4 | Normal |
| NCU07556 | MOT-1 kinase | 210 | 2 | 1 | ND |
| NCU05608 | IP-3 kinase | 285 | 2 | 1.1 | ND |
| NCU03545 | Leucine Rich Repeat protein | 253 | 2 | 1.8 | ND |
| NCU03892 | related to RSC-9 | 115 | 1 | 0.77 | ND |

^1^ The gene identification #’s were obtained from the Neurospora crassa genome database (http://www.broadinstitute.org/annotation/genome/neurospora/MultiHome.html). The rows highlighted in bold indicate members of the DCDC complex characterized in the present manuscript.

^2^ The protein descriptions were obtained from the Uniprot database (http://www.uniprot.org)

^3^ Molecular weight is shown in kilodaltons.

^4^ The # of unique peptides identified by mass spectrometry was determined using Scaffold 2.06 with the minimum peptide probability set to 95%

^5^ The % coverage of each protein by peptides identified by mass spectrometry was determined using Scaffold 2.06 with the minimum peptide probability set to 95%.

^6^ The DNA methylation phenotype was determined for select knockout strains by Southern hybridization (ND = no data).
