# Supplementary material for: DNA Methylation and Normal Chromosome Behavior in Neurospora Depend on Five Components of a Histone Methyltransferase Complex, DCDC
Source: PLoS Genet. 2010 Nov 4;6(11):e1001196. doi: 10.1371/journal.pgen.1001196 (PMC2973830; doi:10.1371/journal.pgen.1001196)
Supplement: Table S3 — Strains used in this study. (0.13 MB DOCX) [file pgen.1001196.s007.docx]

**Table S3. Strains used in this study**

| **Strain** | **Genotype** | **Source/reference** |
| --- | --- | --- |
| **N2977** | *his-3^RIP^::bar^M^::his-3^RIP^; am Δinl ; am^RIP^::hph^M^::am^RIP^ a* | [1] |
| **N3312** | *his-3^RIP^::bar^M^::his-3^RIP^; am Δinl dim-7^UV64^ ; am^RIP^::hph^M^::am^RIP^ a* | [1] |
| **N3407** | *dim-8^193-2^ his-3^RIP^::bar^M^::his-3^RIP^; am Δinl; am^RIP^::hph^M^::am^RIP^ a* | This study |
| **N3926** | *dim-9^222-7^ his-3^RIP^::bar^M^::his-3^RIP^; am Δinl ; am^RIP^::hph^M^::am^RIP^ a* | This study |
| **N3927** | *dim-9^229-4^ his-3^RIP^::bar^M^::his-3^RIP^; am Δinl ; am^RIP^::hph^M^::am^RIP^ a* | This study |
| **N3928** | *hda-1^333-1^ his-3^RIP^::bar^M^::his-3^RIP^; am Δinl ; am^RIP^::hph^M^::am^RIP^ a* | This study |
| **N3929** | *hda-1^193-3^ his-3^RIP^::bar^M^::his-3^RIP^; am Δinl ; am^RIP^::hph^M^::am^RIP^ a* | This study |
| **N3939** | *hda-1^453-1^ his-3^RIP^::bar^M^::his-3^RIP^; am Δinl ; am^RIP^::hph^M^::am^RIP^ a* | This study |
| **N1850** | *Δdim-2* | [2] |
| **N3403** | Δ*dim-8 a* | FGSC #11197 |
| **N3940** | *Sad-1 Δdim-8 A* | This study |
| **N3941** | Δ*dim-8 A* | This study |
| **N3263** | *Δdim-8 ::dim-8-3xHA* | This study |
| **N3942** | *dim-8^193-2^ ::dim-8-3xha; ::Bml* | This study |
| **N3943** | *Δddb-1; hpo-gfp* | This study |
| **N39** | *fl; A* | FGSC# 4317 |
| **N40** | *fl; a* | FGSC# 4347 |
| **N150** | (74-OR23-IV)  *A* | FGSC# 2489 |
| **N623** | *his-3 A* | FGSC# 6525 |
| **N625** | *his-3 a* | FGSC# 6103 |
| **N1877** | *his-3; dim-2; a* | [2] |
| **N2240** | *rid^RIP4^ his-3 A* | [3] |
| **N2257** | *rid^RIP4^ his-3 a* | [3] |
| **N2264** | *his-3; dim-5 leu-2 pan-1 a* | [4] |
| **N2556** | *his-3; hpo^RIP2^ a* | [5] |
| **N2534** | *his-3^+^::hpo^+^-sgfp^+^ A* | [5] |
| **N2542** | *his-3^+^::hpo^+^-sgfp^+^; dim-5 leu-2 pan-1 a* | [5] |
| **N3016** | *Sad-1 his-3 a* | This study |
| **N3414** | *Sad-1 his-3; ddb-1::hph^+^ a* | This study |
| **N3892** | *Sad-1 his-3; cul-4^RIP1^ a* | This study |
| **N3893** | *Sad-1 his-3^+^::cul-4 ^+^; cul-4^RIP1^ a* | This study |
| **N3894** | *Sad-1 his-3^+^::cul-4 ^+^; cul-4^RIP1^ a* | This study |
| **N3895** | *Sad-1 his-3^+^::hpo^+^-sgfp^+^; cul-4^RIP1^ a* | This study |
| **N3896** | *Sad-1 his-3^+^::FLAG-HAT-cul-4^+^; cul-4^RIP1^ a* | This study |
| **N3897** | *Sad-1 his-3^+^::FLAG-HAT-cul-4^+^; cul-4^RIP1^ a* | This study |
| **N3900** | *Sad-1 his-3^+^::FLAG-HAT-cul-4^+^; cul-4^RIP1^ a* | This study |
| **N3901** | *Sad-1 his-3^+^::hH2A^+^-sgfp^+^; cul-4^RIP1^ a* | This study |
| **N3944** | *Δdim-5 A* | This study |
| **N3855** | *Sad-1 Δdim-7::hph^+^ A* | [1] |
| **N3856** | *Δdim-7::hph^+^ a* | [1] |
| **N3169** | *Δcul4::hph a* | FGSC #12374 |
| **N3411** | *Δdim-9 a* | This study; FGSC #15823 |
| **N3412** | *Sad-1 his-3^RIP^::bar^M^-his-3^RIP^;* Δ*dim-9 A* | This study; FGSC #15823 |
| **N3945** | Δ*nfh-1::hph* | FGSC #12725 |
| **N3946** | Δ*nfh-2::bar* | This study |
| **N3947** | Δ*nfh-1::hph;* Δ*nfh-2::bar* | This study |
| **N3902** | *his-3^+^::hH2A^+^-sgfp^+^ a* | This study |
| **N3903** | *his-3^+^::hH2A^+^-sgfp^+^; dim-5 leu-2 pan-1 a* | This study |
| **N3904** | *his-3^+^::hH2A^+^-sgfp^+^; hpo^RIP2^ a* | This study |
| **N3905** | *his-3^+^::hH2A^+^-sgfp^+^; dim-2 a* | This study |
| **N3906** | *Sad-1 his-3^+^::hH2A^+^-sgfp^+^; ddb-1::hph^+^ a* | This study |
| **N3948** | *dim-8-3xflag-hph^+^* | This study |
| **N3949** | *dim-8-3xflag; Δcul4* | This study |
| **N3950** | *dim-8-3xflag; Δdim-9* | This study |
| **N3951** | *dim-8-3xflag; Δdim-7* | This study |
| **N3952** | *dim-9-3xflag-hph^+^* | This study |
| **N3953** | *dim-9-3xflag; dim-5-3xha* | This study |
| **N3954** | *dim-9-3xflag; Δcul4* | This study |
| **N3955** | *dim-9-3xflag; Δdim-8* | This study |
| **N3956** | *dim-9-3xflag; Δdim-7* | This study |
| **N3857** | *dim-7-3xflag-hph^+^* | Lewis *et al.* |
| **N3957** | *dim-7-3xflag; Δcul4* | This study |
| **N3958** | *dim-7-3xflag; Δdim-8* | This study |
| **N3959** | *dim-7-3xflag; Δdim-9* | This study |
| **N3864** | *his-3^+^::dim-5-dam* | [1] |
| **N3865** | *his-3^+^::dim-5-dam; Δdim-7* | [1] |
| **N3960** | *his-3^+^::dim-5-dam; Δcul4* | This study |
| **N3961** | *his-3^+^::dim-5-dam; Δdim-8* | This study |
| **N3962** | *his-3^+^::dim-5-dam; Δdim-9* | This study |
|  |  |  |
|  |  |  |
