# Supplementary material for: DNA Methylation and Normal Chromosome Behavior in Neurospora Depend on Five Components of a Histone Methyltransferase Complex, DCDC
Source: PLoS Genet. 2010 Nov 4;6(11):e1001196. doi: 10.1371/journal.pgen.1001196 (PMC2973830; doi:10.1371/journal.pgen.1001196)
Supplement: Table S4 — Oligos used in this study. (0.13 MB DOCX) [file pgen.1001196.s008.docx]

**Table S4. Oligos used in this study**

| **Name** | **Sequence** |
| --- | --- |
| 1644 | ATTCAGACCCCATTAGCCGTCCACGCC |
| 2193 | CGCTTAAGAGCGCTGACATAGCGATGATGCTT |
| 2194 | GCTCTAGAGCCATTCGTTAGAATCACAATACA |
| 2195 | CTCCCACGCAATTCAAAGCTT |
| 2196 | CTGCTCCCAACAAGCGCTCCC |
| 2197 | GGCTACAGTCCTAGTCCCGGC |
| 2198 | CGCAAATATGCTGACCATCGG |
| 2199 | CGGGTGCGGCCATTGTCAGCG |
| 2200 | AACGAGTGCGGTTCAAGTTTC |
| 2201 | GCGCGTCCTCAACAAACACCC |
| 2206 | GTAACGCCAGGGTTTTCCCAGTCACGACGGGATCCGTGCGTACAGTGGTGCGGAT |
| 2207 | GTGGATGCACGTCTGCCCTGGAGATTGTTGGAGCTCCATCGATGGACAAGTCGTTT |
| 2208 | TGGTGGTTGTTGACCGTGATACAAACTCCTAGCTAGCGTCGCAGTGCTAGCCGTGAC |
| 2209 | TGAGCGGATAACAATTTCACACAGGAAACAGCGAATTCGCTGAAGGGGGTTAAGAATAG |
| 2210 | TCCGGGCCCAGCGCTGACATAGCGATG |
| 2211 | CGGAATTCCGCAAGATAGACATAATT |
| 2216 | GAAGATCTATGCCTTCGAACAGCACT |
| 2217 | GCTCTAGACGCAAGATAGACATAATT |
| 2283 | GAAGATCTATGTCCGCCAAACTCAAG |
| 2284 | GAAGATCTATGGCCACGGGCAAAACA |
| 2285 | GAAGATCTATGGAGAAACTATACCGC |
| 2286 | GTGCGCATCATGGCCAGCCGGAAGAAG |
| 2287 | CTTCTTCCGGCTGGCCATGATGCGCAC |
| 2288 | AGGCGCGCCAATGCCTTCGAACAGCACT |
| 2289 | CCTTAATTAACGCAAGATAGACATAATT |
| 2290 | CGGAATTCAGCGCTGACATAGCGATGATGCTT |
| 2291 | CGGGATCCCTATGGACAAGTCGTTTTCAACAA |
| 2292 | TCCGGGCCCAGCGCTGACATAGCGATGATGCTT |
| 2293 | CGGAATTCGCCGCCTCCGCCGCCCTT |
| 2294 | CGCATCATGGCCAGCCGGAAG |
| 2295 | CTTCCGGCTGGCCATGATGCG |
| 2296 | CGGAATTCCTTAATTAAAGACTACAAA |
| 2297 | GAAGATCTTCCGCCGCCTCCGCCCTTGTCATCGTCATC |
| 2338 | ATAAGAATGCGGCCGCGCCTGATCGCTGATGGGA |
| 2686 | CGGAATTCAAGTTCTTGACTCGCGTT |
| 6605 tag FP | gag gtc gac ggt atc gat aag ctt gat ata tcg tcg gta caa gct tcc t |
| 6605 tag RP | cct ccg cct ccg cct ccg ccg cct ccg ccg tgc atc ctc cgt agc tcc tct a |
| 6605 tag UTR FP | TGC TAT ACG AAG TTA TGG ATC CGA GCT CGA TGA TTG GAT GTC TAC AAG T |
| 6605 tag UTR RP | ACC GCG GTG GCG GCC GCT CTA GGA CTA GTG GTG AGA GCG CGA ACG GC |
| 1656 tag FP | GAG GTC GAC GGT ATC GAT AAG CTT GAT ATA TCA ATG AGC TCA CTG TCA TG |
| 1656 tag RP | CCT CCG CCT CCG CCT CCG CCG CCT CCG CCG AAT TCA TCA ATG TCG CTT CC |
| 1656 UTR tag FP | TGC TAT ACG AAG TTA TGG ATC CGA GCT CGT TTA AGA GTT CTA TGT AAT CAG |
| 1656 UTR tag RP | ACC GCG GTG GCG GCC GCT CTA GAA CTA GTA ACG AAG CGT GGC ATC GAG CT |
| INL INV1 | GAC CCT TGA GCA CAA GCT C |
| INL INV2 | TTG GTG CCG TTG ATA CCG |
| pRATT seq2 | GCA AGA GCA ACT CGG TCG CC |
| Rescue FP | CAGGAAGGCAAAATGC |
| Rescue RP | TAGGTGTACTTGGAGCG |
| NCU06605 NotI FP | GTA AGC GGC CGC GGC TTG CTC ATG GTC GCT GTC CAC CG |
| NCU06605 PacRP | GGC CTT AAT TAA GTG CAT CCT CCG TAG CTC CTC TAC CAT |
| 2806 KO 5' FP | GTA ACG CCA GGG TTT TCC CAG TCA CGA CGT GTA TGA TTC CAG GTT GG |
| 2806 KO 5' RP | gct ttt gtt ccc ttt agt gag ggt taa tta aga agc agg aaa tgg ac |
| 2806 KO 3' FP | GCT CCT TCA ATA TCA TCT TCT GTC GAC GGA GAT CTC GAG CAG ACA CT |
| 2806 KO 3' RP | GCG GAT AAC AAT TTC ACA CAG GAA ACA GCC CTC TAC CTC CAA CAG CC |
