# Supplementary material for: DNA Methylation and Normal Chromosome Behavior in Neurospora Depend on Five Components of a Histone Methyltransferase Complex, DCDC
Source: PLoS Genet. 2010 Nov 4;6(11):e1001196. doi: 10.1371/journal.pgen.1001196 (PMC2973830; doi:10.1371/journal.pgen.1001196)
Supplement: Text S1 — Supplemental experimental procedures. (0.11 MB DOCX) [file pgen.1001196.s009.docx]

# Text S1. SUPPLEMENTAL EXPERMENTAL PROCEDURES

*Antibodies*

Western blots and co-immunoprecipitation experiments were performed using the following antibodies: α-FLAG (F3165, Sigma), α-HA (mouse; University of Oregon monoclonal facility or rat; clone 3F10, Roche), α-DIM-5 (provided by X. Cheng, Emory University)[6], α-H3 (Abcam #1791-100), α-H3K4me2 (Upstate (07-030), α-H3K9me3 (a gift from Dr. Prim Singh [7], α-H3K4me3 (Abcam, # ab8580), α-K27me2 (a gift from Dr. Thomas Jenuwein), H3K27me3 (Upstate #67-449), α-H3K36me1 (Abcam ab9084), α-H3K36me3 (Upstate 07-274), α-H3K36 (a gift from Dr. Brian Strahl [8]), α-H3K79me2 (Upstate # 07-366), α-H4K20me3 (a gift from Dr. Thomas Jenuwein), and α-H4Ac4 (Upstate #06-866).

*Selection and identification of dim* mutants

To select for *dim* mutants, approximately three hundred transformations were carried out followed by selection for basta-resistant strains. Approximately 1x10^7^ conidia of strain N2977 were transformed using a plasmid containing a copy of the *inl* gene (pRATT09; provided by Dr. Rodolfo Aramayo, Texas A&M University), which had been linearized with *NdeI*. The transformation mixture was used to inoculate a 125ml flask containing 25mls of medium lacking inositol to select for *inl^+^* prototrophs (1X Vogel’s Salts, 1.5% Sucrose, 1.5% Agar, 1mg/ml alanine, 1mg/ml histidine), and the cultures were allowed to conidiate “en masse” to allow dilution of pre-existing DNA methylation. Approximately 1x10^6^ *inl*^+^ conidia from each transformation mixture were plated on medium containing basta [Vogel’s (-NH_4_NO_3_), 0.5% proline, 1.5% Agar, 50ng/ml inositol, 1mg/ml alanine, 1mg/ml histidine, 2% sorbose, 0.05% glucose, 0.05% fructose, 400mg/ml basta] [9] to select for strains that had reactivated the methylated *bar* gene. Basta-resistant colonies were isolated, transferred to slants containing 400µg/ml basta and tested for growth on 200µg/ml hygromycin. Strains that were resistant to both drugs were analyzed further.

For the *dim-8^193-2^* mutant, the integration site of the *inl* cassette was determined by inverse PCR. Genomic DNA was first digested with *PstI,* which cuts once within the pRATT09 plasmid. Digested DNA was then incubated with T4 DNA ligase (NEB) to generate circular ligation products. Following ligation, inverse PCR was performed using primers INL INV1 plus INL INV2, the ligation mixture as a template, and LA Taq polymerase (Takara) to generate a linear DNA fragment containing DNA from the insertion site. To identify the plasmid insertion site, DNA sequencing reactions were performed using the linear PCR product as a template and the BLA EXT primer.

Although the insertion cassette and the Dim^-^ phenotype of *dim-9* strains were genetically separable, analyses of progeny from a backcross revealed that the insertion cassette and *dim-9^227-2^* were linked, residing ~2 map units apart. We used plasmid rescue to determine that the insertion cassette had integrated on LGII. Genomic DNA was digested with *EcoRI*, which does not cut within the pRATT09 insertional vector. Digested DNA was then incubated with T4 DNA ligase (NEB) to generate circular ligation products and ligation mixtures were transformed into *E. coli* to obtain ampicillin-resistant colonies. We next sequenced across the *NdeI* site to identify the insertion site using Rescue FP and Rescue RP primers.

# *Generation and complementation of cul-4^RIP1^*

# The *cul-4^+^* gene (NCU00272.2), with its native promoter and the downstream regions, were amplified using primers 2206 and 2209 from cosmid pMOcosX5E8 [10]. The PCR product was cloned between the *Spe*I and *Eco*RI sites of pBM61 to generate pKA67. This plasmid was digested with *Dra*III and integrated into the *his-3* locus of strains N623 and N3016 [11]. Two His^+^ transformants of opposite mating types, each containing a duplication of *cul-4* gene, were then crossed to each other to induce RIP. Six slow growing and sparsely conidiating progeny were selected for further analysis as putative *cul4* mutants. Analysis of the *cul-4* gene from these strains by PCR-RFLP using the restriction enzyme *Mse*I revealed evidence of extensive mutations by RIP. One of these strains was further crossed to a *his-3* strain (N3016) to obtain strain N3892, which lacks the duplication. DNA sequencing (primers 2195-2201) of the *cul4* allele (*cul-4^RIP1^*) amplified (primers 2206 & 2209) from this strain revealed numerous premature termination codons and other mutations. To test for complementation of *cul-4^RIP1^* with the wildtype allele, plasmid pKA67 was digested with *Dra*I and introduced into the *his-3* locus of strain N3892 by transformation. Two His^+^ transformants (N3893 and N3894) that displayed complementation of DNA methylation (data not shown) were selected for study.

*Cul-4 constructs*

To purify proteins associated with CUL-4, we made a construct (pKA122) to express this protein with N-terminal tags consisting of Met-FLAG-HAT-3xFLAG and 5x-glycine spacers between the epitopes and between them and CUL-4. Because there was uncertainty about which methionine codon represented the actual start of the protein, we transformed strain N3892 with four constructs to test the function of four potential start codons and tested the transformants for defects in DNA methylation. Methionines were labeled M1, M2, M3, and M4, based on the predicted amino acid sequence of the prediced protein encoded by NCU00272 (XP_957743.2). N-terminal, FLAG-tags introduced at methionine #1 (MPSNS), methionine #2 (MSAKL), and methionine #3 (MATGK) all complemented the DNA methylation defect, whereas an N-terminal, FLAG tag introduced at methionine #4 (MEKL) failed to restore DNA methylation in the *cul4* mutant.

*DDB1 and DIM-9 knock-in strains*

Fragments of the DDB1 coding sequence and the DDB1 3’UTR were amplified with 6605 tag FP plus 6605 tag RP and 6605 tag UTR FP and 6605 tag UTR RP, respectively. Fragments of the DIM-9 coding sequence and the DIM-9 3’ UTR were amplified with 1656 tag FP plus 1656 tag RP and 1656 UTR tag FP plus 1656 UTR tag RP, respectively. Knock-in plasmids were assembled by yeast in vivo recombination with a 10XGly-3XFLAG-hph fragment and pRS416 as described [12]. Southern blots were performed to verify that all knock-in constructs support wildtype levels of DNA methylation.

*H2A-GFP* constructs

To facilitate tracking chromatin cytologically, we engineered a GFP-tagged version of *Neurospora crassa* histone H2A. The *hH2A* gene (NCU02437.2), including its native promoter, was amplified from wildtype genomic DNA (strain N150) with primers 2388 and 2686 and cloned into the GFP-experssion vector, pCCG::C-Gly::GFP [12]. The resulting plasmid (pKA134) was digested with *Xmn*I and integrated at the *his-3* locus of various strains by electroporation to generate His^+^ transformants that express H2A-GFP in various genetic backgrounds (Table S3). To facilitate tracking chromatin cytologically, dilute conidial suspensions of wildtype and mutant strains expressing H2A-GFP were spotted on agar medium (Vogel’s N medium containing 0.5% sucrose and supplemented with 0.2 mg/ml leucine and 0.01 mg/ml pantothenic acid) and incubated at 32°C. Square pieces of agar with germinating conidia (after 2hrs of growth) or hyphae (after overnight growth) were cut with glass coverslips and placed on glass slides. These agar pieces were flooded with liquid Vogel’s N medium and covered with coverslips for visualization of H2A-GFP.

REFERENCES

1. Lewis ZA, Adhvaryu KK, Honda S, Shiver AL, Selker EU (2010) Identification of DIM-7, a protein required to target the DIM-5 H3 methyltransferase to chromatin. Proc Natl Acad Sci U S A 107: 8310-8315.

2. Kouzminova EA, Selker EU (2001) *Dim-2* encodes a DNA-methyltransferase responsible for all known cytosine methylation in Neurospora. EMBO Journal 20: 4309-4323.

3. Freitag M, Williams RL, Kothe GO, Selker EU (2002) A cytosine methyltransferase homologue is essential for repeat-induced point mutation in Neurospora crassa. Proc Natl Acad Sci U S A 99: 8802-8807.

4. Tamaru H, Selker EU (2001) A histone H3 methyltransferase controls DNA methylation in Neurospora crassa. Nature 414: 277-283.

5. Freitag M, Hickey PC, Khlafallah TK, Read ND, Selker EU (2004) HP1 is essential for DNA methylation in Neurospora. Mol Cell 13: 427-434.

6. Collins RE, Tachibana M, Tamaru H, Smith KM, Jia D, et al. (2005) In vitro and in vivo analyses of a Phe/Tyr switch controlling product specificity of histone lysine methyltransferases. J Biol Chem 280: 5563-5570.

7. Tamaru H, Zhang X, McMillen D, Singh PB, Nakayama J, et al. (2003) Trimethylated lysine 9 of histone H3 is a mark for DNA methylation in Neurospora crassa. Nat Genet 34: 75-79.

8. Adhvaryu KK, Morris SA, Strahl BD, Selker EU (2005) Methylation of Histone H3 Lysine 36 Is Required for Normal Development in Neurospora crassa. Eukaryot Cell 4: 1455-1464.

9. Pall ML (1993) The use of Ignite (Basta;glufosinate;phosphinothricin) to select transformants of bar-containing plasmids in Neurospora crassa. Fungal Genetics Newsletter 40: 58.

10. Orbach MJ (1994) A cosmid with a HyR marker for fungal library construction and screening. Gene 150: 159-162.

11. Margolin BS, Freitag M, Selker EU (1997) Improved plasmids for gene targeting at the his-3 locus of Neurospora crassa by electroporation. Fungal Genetics Newsletter 44: 34-36.

12. Honda S, Selker EU (2009) Tools for fungal proteomics: multifunctional neurospora vectors for gene replacement, protein expression and protein purification. Genetics 182: 11-23.
